# Cysteine Cathepsin Protease Inhibition: An update on its Diagnostic, Prognostic and Therapeutic Potential in Cancer

**DOI:** 10.3390/ph12020087

**Published:** 2019-06-11

**Authors:** Surinder M. Soond, Maria V. Kozhevnikova, Paul A. Townsend, Andrey A. Zamyatnin

**Affiliations:** 1 Institute of Molecular Medicine, Sechenov First Moscow State Medical University, Trubetskaya str. 8-2, 119991 Moscow, Russia; 2 Federal State Autonomous Edu­cational Institution of Higher Education I.M. Sechenov First Moscow State Medical University of the Ministry of Healthcare of the Russian Federation (Sechenovskiy University), Hospital Therapy Department No. 1, 6-1 Bolshaya Pirogovskaya str, 119991 Moscow, Russia; Kozhevnikova-m@inbox.ru; 3 Division of Cancer Sciences and Manchester Cancer Research Centre, Faculty of Biology, Medicine and Health, University of Manchester, Manchester Academic Health Science Centre, and the NIHR Manchester Biomedical Research Centre, Manchester M20 4GJ, UK; paul.townsend@manchester.ac.uk; 4 Institute of Molecular Medicine, Sechenov First Moscow State Medical University, Trubetskaya str. 8-2, 119991 Moscow, Russia; 5 Belozersky Institute of Physico-Chemical Biology, Lomonosov Moscow State University, 119992 Moscow, Russia

**Keywords:** cathepsin, cystatin, cancer, diagnosis, prognosis, chemotherapy, theranostics

## Abstract

In keeping with recent developments in basic research; the importance of the Cathepsins as targets in cancer therapy have taken on increasing importance and given rise to a number of key areas of interest in the clinical setting. In keeping with driving basic research in this area in a translational direction; recent findings have given rise to a number of exciting developments in the areas of cancer diagnosis; prognosis and therapeutic development. As a fast-moving area of research; the focus of this review brings together the latest findings and highlights the translational significance of these developments.

## 1. Cathepsin Expression and Regulation

Recently, the cathepsin family of proteases have gained significant attention particularly in the field of cancer biology due to their ability to modulate the architecture of the Extracellular Matrix (ECM, thus promoting tumor dispersal) [1,2].

Together, the 15 lysosomal proteins that make up the human family of cathepsin proteases, which can be broadly classified into aspartic (D, E)-, serine (A, G)- and cysteine (B, C, F, H, K, L, O, S, V, Z/X, W)-proteases types [3]. Additionally, such proteases can be classified further based on their proteolytic activity as endo-peptidases (S, K, V, F, L) and both endo- and exo-peptidase (B, H, Z/X, C). Some of the cathepsins also have broad tissue-specific expression, such as cathepsins C, B, H, K, O, L and Z, and studies of which have revealed general mechanisms for substrate cleavage, lysosome research and cell death [4].

Taken with the observations that some of these proteases are found to show upregulated levels of expression in cancer progression, it comes as no surprise therefore that some of them have been validated as potential therapeutic targets in normal developing cancer types and cancer types that show resistance to regular chemotherapeutic regimes. Of significance are the cathepsins which are over-expressed in cancers that show biological features consistent with late stage cancer and tumor metastases [5]. However, juxtaposed with this feature of the cathepsins is their importance in inducing lysosome-derived apoptosis by activating the caspases (through mitochondrial outer membrane permeabilization [6]) in most cancer types [7]. When taken with the observations that some members of this family of 15 proteases show pleiotropic activity towards common substrates [8], it does therefore make the task of designing therapeutics against cancer-specific cathepsins more arduous that expected. Nevertheless, this obstacle has been partially addressed through validating engineered peptides in active site mapping approaches and which have yielded some very encouraging small molecule therapeutics [9,10,11,12].

As the expression of certain cathepsins are significantly upregulated in certain cancer types it has also been reported that their ablation has the potential to sensitize cancer cells to conventional chemotherapeutic treatments. For example, this effect has been seen in the instances of Cathepsin L over-expression in ovarian and lung cancer cells which offers a key perspective that can be exploited for the purpose of therapeutic design [13,14,15]. Moreover, this may also offer great potential for the design of robust diagnostic and prognostic assays.

**Figure 1:**
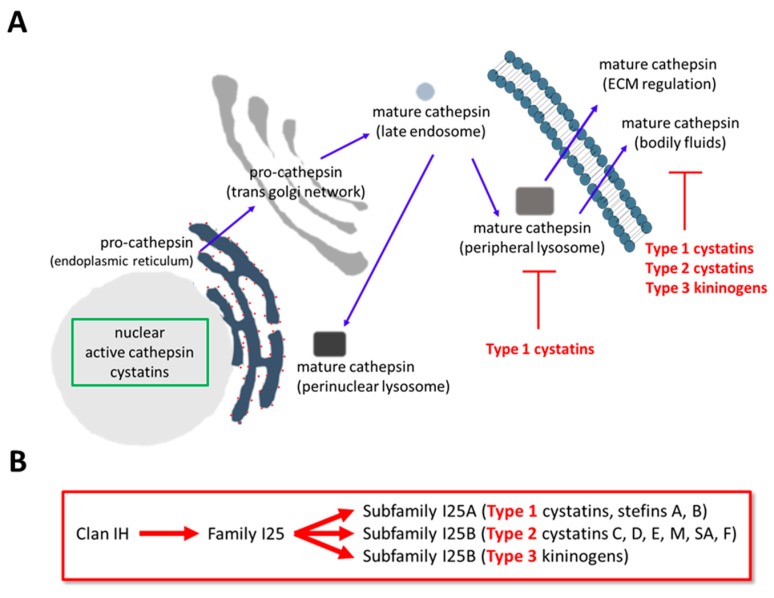
(**A**) Cathepsin protein synthesis and localization. Cathepsins are synthesized and enter the secretory pathway as inactive zymogens. Mature cathepsins arise from the removal of their pro-domains in the endosomes after which they are transported to the perinuclear lysosomes (in normal cells) or peripheral lysosomes (in cancer cells) after which they can be secreted into the extracellular space. The localization of inhibitory-cystatins and -kininogens are highlighted as is the ability of cathepsins and cystatins to become localized in the nucleus (green box). (**B**) Classification of cystatin family members. In this classification, the cystatin inhibitors can be arranged into clans, families, sub-families and type.

Broadly, the cathepsin family range in 50 to 90 KDa in size which after translation are inserted into the endoplasmic reticulum (ER), where they enter the secretory pathway as inactive zymogens. Within the ER, they are post-translationally modified and following their transport through the trans-golgi network, become trans- and auto-activated upon reaching the acidic environment of the endosomes [16,17,18]. Once activated, the cathepsins can be transported to the lysosomes (as in normal cells) or then secreted as in most cancer cells (Figure 1A).

All cysteine cathepsins can be regulated by the naturally-occurring endogenous cystatin inhibitors (Figure 1A). This key step may also modulate the invasiveness of cells aberrantly expressing the cathepsin proteins [19]. There are four main groups of cathepsin inhibitors (for an excellent review article, see [20]), based upon protein sequence and structural similarities, the stefins, cystatins, kininogens and non-inhibitory homologues of cystatins [21] (such as fetuins [22,23]), which can also be arranged into clans, families and sub-families (Figure 1B). The stefins (or type 1 cystatins) are approximately 100 amino acids (aa) in length, are produced mainly intracellularly [24] and can also be found secreted into extracellular spaces [25].

The cystatins (or type 2 cystatins) are approximately 115 aa in length, are secreted and the group members characteristically containing two conserved disulphide bonds [26]. The kininogens make up the group of type 3 cystatins and are found secreted into the blood and of which there are three types-low molecular weight kininogens, high molecular weight kininogens and the T-kininogens [27]. Using an alternative nomenclature, cystatins have also been classified based upon subfamilies and family members of Clan IH (see Figure 1B).

How these inhibitor proteins exert their activity in disease through the modulation of specific cysteine cathepsins (Table 1) has been evidenced over the recent years through structural studies and has generated a great deal of therapeutic interest [21]. While the cystatins do broadly offer therapeutic potential, they are however limited in their therapeutic uses due to the broad specificity and redundancy with which they inhibit the cathepsin family members. Briefly, cystatins and stefins bind each cathepsin type, with high affinity and are reversible in their inhibition [27]. Mechanistically, the general consensus is that the cathepsin-bound inhibitory cystatins are released from the active cathepsin upon substrate recognition [21]. As seen from the chicken cystatin [28] and human stefin B [29] structural studies, three binding sites exist between the cystatin and its cognate cathepsin protease and each of which is made up of conserved aa sequences. Furthermore, co-crystallization studies between stefin A and cathepsin B [30], have revealed key residues that are essential for substrate/inhibitor binding, hydrolysis [31] and how stefin A is consequently capable of discriminating between endopeptidases (cathepsin L and S) and exopeptidases (cathepsin B, C and H) [32]. When taken with the recent advancements in defining substrate cleavage sites using peptide library screening, a clearer picture has therefore emerged with regards to how these inhibitors may modulate cathepsin-substrate specificity and to what extent [33,34]. Based on the relationship between cystatin expression and cathepsin activation, it therefore comes as no surprise that while utilizing the cathepsins as diagnostic (or prognostic) markers, we must simultaneously bring the cystatins into focus as well. This aspect offers greater accuracy in determining how catalytically active the overexpressed cathepsins may be and therefore what contribution they can make to cancer progression in vivo. Moreover, based on outcomes from substrate cleavage site and peptide screening studies, an alternative approach to quantifying and targeting active cathepsins has also evolved in the form of ‘theranostics’.

In this review, we evaluate recent advancements in the use of these various approaches and the potential they hold in quantifying active cathepsin proteases for diagnostic, prognostic and therapeutic purposes.

## 2. Transcriptional Regulation of Cystatins in Cancer

More recently, the transcriptional and post-translational regulation of the cystatin family members have gained greater importance, due to their key regulatory effects on cathepsin protein activity and because they hold good potential in diagnostic and prognostic assay design. Additionally, they have also been found to modulate nuclear gene expression, while at the same time their genes are amenable to transcriptional regulation upon cell cycle arrest. For example, Mori et al. (2016) found cystatin C transcription to be negatively regulated by p53 in response to DNA damage and a lower expression level of cystatin C suggested to offer a poor prognosis in breast cancer (BC) [35]. Cystatin A expression (which inhibits cathepsins B, H and L) was down-regulated in lung cancer cell lines and over-expressed cystatin A inhibited cathepsin B-mediated colony formation, migration and invasion [36,37]. Interestingly, colorectal cancer (CRC) cells which lacked stefin B, gave rise to enhanced cathepsin L activity and its nuclear localization with the effect of accelerating cell cycle progression in HCT 116 cells [38]. Similarly, a portion of cystatin D had also been observed to reside in the nucleus of colon carcinoma (CC) cells where it altered the transcription of a number of tumor-related cytokines such as FGF4 and CXC3CL1 [39]. Collectively, such findings highlight the ability of cystatin proteins to take on nuclear-related functions. Mechanistically, how cystatins are trafficked to the nucleus remains largely undefined, whereas nuclear cathepsins may arise due to the intracellular expression of cathepsin proteins that lack a secretion signal [40] due to possible alternative splicing of mRNA transcripts [41].

Collectively such findings highlight the on-going importance of characterizing cystatin gene and protein expression, clearly as their ability to naturally inhibit aberrant cathepsin activity has implications in the clinical setting. Their input potentially offering greater effectiveness of chemotherapy, particularly in the context of tumor proliferation, chemotherapeutic resistance and tumor invasion.

## 3. Cathepsins and Cystatins as Diagnostic Markers for Cancer

Recently, significant emphasis has been placed on the use of quantified cathepsin (and/or cystatin expression ratios) in developing novel and more accurate diagnostic patient tests. These are aimed at measuring more precisely these expression products from tumor cells in the absence of any possible interference from ECM-derived stromal cells [42]. Such tests have been reported with great diversity and with varying degrees of complexity. As far back as 2016, diagnostic tests for cathepsin B and D were published for nasopharyngeal carcinoma by measuring serum cathepsin protein levels using ELISA [43]. Here, the study comprised 80 disease samples and although cathepsin B could be measured with relatively low sensitivity (61.9%) with 63.2% specificity (at 12.4 mg/L for, Receiver Operating Characteristics (ROC) analysis), cathepsin D could be measured with greater accuracy with 66.7% sensitivity and 58.5% specificity (at a cut off concentration of 14.7 mg/L for ROC analysis). Collectively, while such findings highlighted the validity of such diagnostic approaches for some cathepsin family members, they do emphasize limitations for others.

Subsequently, Liu et al. (2016) [44] measured serum cathepsin S protein from 496 gastric cancer patients, using ELISA tests and described an assay with 60.7% sensitivity and 90.8% specificity (at an Area Under the Curve (AUC) of 0.803). This could also be correlated with tumor volume, node status, tumor stage, metastasis and prognosis [44]. Meanwhile, Kehlet et al. (2017) [45] also quantified cathepsin S-degraded Decorin from serum samples taken from lung cancer patients and developed a promising assay with a diagnostic power of 0.96 in disease patients (against 0.77 from healthy individuals). Thus, demonstrating an indirect method for the quantification of cathepsin S activity as a diagnostic tool [45].

To compliment such strategies, the cystatin inhibitor proteins have also been incorporated as diagnostic biomarkers in cancer biology. For example, Komura et al. (2017) observed higher cystatin A protein levels in the serum from 36 pancreatic ductal carcinoma (PDAC) patients in comparison to normal patients [46]. Additionally, Tokarzewicz et al. (2018) measured cystatin C from the serum of 90 bladder cancer patients which was and observed values (0.35 +/− 0.02 g/ml) significantly lower than healthy individuals (0.68 +/− 0.05 g/ml) with a sensitivity of 87% and specificity of 92% achieved at a concentration of <0.54 g/ml cystatin C [47]. As such opposing levels in cystatin expression were observed, suggestive of conflicting roles and functions for the cystatins, further development of assays for cystatins may be required for accurately evaluating each of these cancers types [48,49].

Consequently, attempts have been made to offer a more robust assay which paints an accurate picture of how potentially active specific cathepsin levels may be derived from disease patients. Therefore, recent assays have been developed to simultaneously quantify the levels of serum cathepsin and cystatin expression. Here, Zhang et al. (2017) quantified cathepsin B and cystatin C ratios from 95 patients with CRC using a variety of control samples and observed statistically significantly higher ratios in disease patients in comparison to normal [50]. In a similar approach, Yan et al. (2017) measured cystatin C and cathepsin B levels in serum from 56 esophageal cancer patients and found values of 703.96 +/− 23.6 ng/ml for cystatin C and values of 38.35 +/− 4.3 ng/ml for cathepsin B in disease subjects and related these values to lymph node metastasis. While the findings showed promise, of importance here was also the ability of this assay to be correlated with lymph node metastasis and thus offering a test with good prognostic [51].

To compliment and develop such studies, alternative (yet synergistic) avenues of investigation that appear to be receiving much attention include the development of activity-based probes (ABPs) in theranostic-based assays. While such assays have the potential to measure directly the catalytic activity and subcellular localization of specific cathepsins, they also having huge potential in being able to be adapted for cathepsin targeting and therapeutic delivery. Initially, such ABPs were designed to bind cathepsin proteins in an activity-dependent manner [52] but more recently, they have been engineered to incorporate quench-fluorescent ABPs (qABPs). Such probes have spawned a new way of investigating the cathepsins for basic research purposes or their utilization in the clinical setting. For example, one of their strengths is permitting the visualization of active cathepsin proteins during real-time imaging in living cells and non-invasively in whole animals [53,54,55]. The enormous potential of such technology is reflected by how fast this area of cathepsin research is currently evolving. For example, the power of ABP technology has been harnessed in photodynamic therapy after coupling the ABP with a photosensitizer (PS-ABP) which could be used to target and induce apoptosis of tumor associated macrophages expressing cathepsins B, L and S [52]. Alternatively, ABPs and qABPs have been developed for detecting cathepsin activities from a diverse range of pathological conditions, such as osteoarthritis (cathepsins B, L and S [56]), inflammation (cathepsin S [57]), idiopathic pulmonary fibrosis (cysteine cathepsins [58]), Atherosclerosis [59], breast cancer (cathepsins X, B, S and L [60]) and lung cancer [61]. Generally speaking, ABPs, qABPs and PS-ABPs therefore offer an alternative for specific detection of active cathepsins through relatively simple and quick quantification techniques through cathepsin labeling, in vitro staining of biopsy cells and even live cell imaging in vivo (see Table 2).

Collectively, there has been positive progress reported using a variety of methods quantifying cathepsins, expressing their levels as a ratio of cystatin to cathepsin enzyme activity in vivo, and using a variety cohort sizes, samples or approaches (see Table 3 for additional articles). When we consider the nuclear localization of cathepsins L, B and D [38,65,66] or cystatin D and B [39,67] proteins, it is worth bearing in mind that the levels of such proteins detected in the serum may not necessarily correspond to actual expression levels observed at the cellular level. Moreover, we must also consider this with the observation that some cystatins can be degraded by cathepsins under acidic conditions [68] and so some of these approaches (Table 2) may offer limitations. Consequently, the analysis of sera samples for cathepsin activity (using ELISA-based methods) may still have some dependency on immunohistochemistry-, mRNA quantification analysis- or even ABP-based assays for greater accuracy.

## 4. Cystatins and Cathepsins as Prognostic Markers in Cancer

As key mediators of cancer progression, the cathepsins of late have also received much attention as potential prognostic markers in developing assays incorporating tumor size, grading, oestrogen receptor expression and lymph node diffusion [69]. Here, a number of excellent studies have recently been published from a number of groups and the driving force behind such studies has revolved around the emergence of chemoresistance (as seen with triple negative BC cells) and a genuine requirement for robust prognostic testing [70,71]. Based on the knowledge that enhanced intracellular cathepsin expression may be correlated with metastatic potential in cancer cells, current lines of research in this area do show great promise. This is clearly demonstrated through the diversity of prognostic assays that have been published over the last three years. While an inverse relationship generally exists between cystatin abundance within the tumor microenvironment and the stage of progression of the cancer, this relationship has not always been fully observed. For example, decreased levels of cystatin C have been correlated with better survival rates, as seen in a recent study for the prognosis and evaluation of renal cell carcinoma patients [72], while conversely enhanced levels of cystatin C were observed in patients with melanoma and CRC [48].

Recently, assays have incorporated cathepsins B, D, G, K, L, V and S quantification using IHC (from BC patients [73]), RT-PCR, Western analysis, reverse-phase protein arrays (from acute lymphoid leukemia patients, ALL [74]), cathepsin expression levels and activity analysis (from acute myeloid leukemia patients, AML [75]), in silico analysis and histological staining (from papillary thyroid cancer patients [76] and esophageal cancer patients [51]). All of these approaches look very encouraging and have offered high levels of accuracy and which highlight the therapeutic-targeting potential of cathepsins and their use as reliable prognostic markers [77,78]. However, to avoid controversy stemming from the cell types from which the quantified cathepsin proteases may originate (stromal, tumor or stem cells, for example [79]), future studies may have to be tailored with greater specificity. This could be addressed by testing different patient subgroups at different stages of disease progression. Such studies may also address the potential for disease relapse and is an area that has been developed further in this context by Guerra et al. (2016) [69]. Here the tumor suppressor p53, cathepsin D and Bcl-2 expression levels were assayed jointly as prognostic markers in BC cells with very encouraging outcomes [69].

## 5. Cathepsin-Derived and -Targeted Therapeutics

One of the most exciting areas in cathepsin research that has enjoyed a flurry of activity of the last 3 years is the development and publication of therapeutics, either directed at the cathepsins or derived from them. A wealth of knowledge has been published in mapping the active sites for the cathepsins and thus given rise to a number of peptides and inhibitors as candidate therapeutics [34]. While in some ways the development of immunotherapeutic products may offer popular alternatives, the lack of tumor-specific target antigens does offer limitations. Consequently, small molecular inhibitors have taken on greater significance in the context of providing more cost-effective safeguards against disease and relapse. Of note, such inhibitors offer a more flexible therapeutic index, have the ability to target stem cells and tumor cells and possess varying lifespans, specificity and penetrability. Initially this area of therapeutic development had been hampered, due to the high structural similarities between the cathepsins and their active sites [80] which gave rise to problems in targeting individual cathepsins selectively with specific therapeutics. However, the recent focus of therapeutic designs specific for cathepsins B, D, H, L and S demonstrate that such problems can be overcome and have been reported with promising outcomes [81].

In the case of cathepsin B [82], a cathepsin B-derived cleavage site was successfully utilized with a p14^ARF^-derived peptide fused to a polypeptide containing a cell entry signal as a drug. This showed very promising outcomes as a therapeutic against a variety of cancer cell types. Alternatively, Kramer et al. (2017) designed a highly penetrative ‘DARPin’ to successfully target cathepsin B activity in mouse BC models with a K_i_ of 35 picomolar [83]. In a novel approach, Raghav et al. (2017) isolated non-peptide synthetic compounds and successfully tested them against cathepsin -B, -H and -L activity at K_i_ concentration as low as 10^−10^ M [84]. Moreover, Liang et al. (2018) used cathepsin B-Doxorubicin cleavable constructs to improve efficacy of therapy in their models to 1.4–1.7 fold over using Doxorubicin alone in B16 Melanoma cells and tumor bearing mice. In this instance, the therapeutic consisted of a Doxorubicin-integrin-specific peptide, separated with a Cathepsin B-cleavable peptide sequence which was assembled onto nanoparticles [85]. Finally, Shim et al. (2018) used carrier-free nanoparticles containing a cathepsin B-cleavage peptide-Doxorubicin prodrug to induce cytotoxicity in HT-29 human colon adenocarcinoma tumor-bearing mice with little toxic side effects [86].

In the case of cathepsin D, small molecule inhibitors against this were used to sensitize triple-positive and -negative BC cells with levels of inhibition exceeding 50% (at 25 M) [87]. Thus, simultaneously highlighting the importance of lysosomal cathepsin D inhibition in sensitizing resistant cells to therapy [88,89,90,91]. Furthermore, Yuan et al. (2018) explored naturally occurring products towards cathepsins -L and -S inhibition and found a compound that worked to varying (yet promising) degrees with an IC_50_ as low as 13.12 μM in the inhibition of BC metastasis [92].

Collectively, all studies show great promise in treating cathepsin-mediated cancer to varying degrees and efficacies in model systems and clearly good progress is being made in defining cathepsin-specific inhibitors with enhanced efficacy of action. Moreover, recent studies clearly demonstrate the use to which cathepsin-specific cleavage sequences or cathepsin-derived sequences can be utilized in targeting tumors more effectively. Further studies addressing how effective these are in animal, translational or clinical contexts by modulating conventional chemotherapeutics to be more effective against resistance are eagerly awaited.

## 6. Clinical Implications and Applications

Based on the articles cited herein, the area of diagnostics holds great promise for designing a cathepsin-based assay that is quick, accurate and reproducible for the diagnostics of gastric, lung, pancreatic bladder, colorectal and esophageal cancers. Simultaneously, such assays may offer great insight into how bio-active secreted or intracellular specific cathepsin proteins may be, particularly in the development of ABPs. As intracellular compartmentalization of some cathepsin and cystatin proteins can potentially affect their observed ratios in serum, there are limitations in using assays that exclusively analyze serum levels alone. Thus, highlighting the importance of incorporating tumor biopsy analysis using IHC (or even cell staining techniques with ABPs) with ELISA assays on sera to ascertain cathepsin/cystatin expression ratios (as shown in Table 2).

From the perspective of therapeutics development, the general findings look very encouraging for targeting extracellular cathepsin proteins and studies have started addressing their efficacy in an approaches that utilizes combined chemotherapeutic treatment in model laboratory systems. This work is indeed at the stage where it can transition into clinical models. For example, how effective such cathepsin therapeutics maybe in a combined therapeutic setting (such as with Tocilizumab [93] or Rituximab [94]), is yet another important application of the above research and the outcomes for which are eagerly awaited. The observations that Tocilizumab or Rituximab also dampen signaling transduction pathways which otherwise activate intracellular cathepsin production (for example IFN- and IL-6 signaling [95,96]), could also be an important consideration. Moreover, ascertaining any incidence of stage-specific resistance, disease relapse, clearance rates and further analysis of any toxic side-effects from these inhibitors may also be important considerations.

## 7. Conclusions

While great strides have been made in the area of cathepsin assay design for diagnostic and prognostic uses, future studies hold great potential. It would appear that at this juncture, we are experiencing a ‘period of transition’ that is being quickly transformed by harnessing the rapidly evolving potential of cathepsin theranostics. When taken together, the future for cystatins and cathepsins in developing cancer research from ‘bench to bedside’ appears to be progressing in good balance and for which the future looks very rewarding.

## Figures and Tables

**Table 1:** Schematic showing members of the cathepsin family and their cognate cystatin inhibitors. As seen towards some serine- and aspartate-cathepsins, no cystatin members are reported to show any inhibitory activity (-).

Cathepsin	Cystatin
Cysteine-	-
B	A, B, C, S
C	S, F
F	F
L	A, B, C, D, E, M, F
H	A, B, C, D, F
K	F
S	B, C, D, F
V	E, M, F
Serine-	
A	-
G	-
Aspartate-	
D	C
E	-

**Table 2:** A selection of recently published diagnostic tests for cathepsins and cystatins. Such tests can be based on quantifying (underlined) mRNA and protein levels from sera using Enzyme Linked Immunosorbent Assay (ELISA) or biopsy samples using Immunohistochemistry (IHC), as denoted by ‘X’. B/D/C-cystatin/stefin denotes measurement of cathepsin (B, D or C) as its ratio to cystatin (Cys) or stefin (Stef) expression in specific disease types.

Cathepsin	mRNA	Protein	ELISA	Biopsy	Serum	Cancer [Reference]
S		X	X	X		Gastric [44]
B/D-Stef	X	X		X		Hepatocarcinoma [62]
B/D-Cys		X			X	Colorectal [50]
K		X		X		Ovarian [63]
Cys A	X	X	X		X	Pancreatic [46]
B/Cys C		X	X		X	Esophageal [43]
X	X	X		X		Glioblastoma [64]

**Table 3:** Cathepsin-specific Activity-Based Probes (ABPs) and qABPs (quench-fluorescent Activity-Based Probes). Positively characterized theranostic probes (Y) with good potential in cathepsin labelling experiments, cell imaging analysis and in vivo imaging are highlighted. NA denotes ‘Not Addressed’.

Theranostic	Cathepsin Specificity	Cathepsin Labelling	Cell Imaging	In Vivo Imaging	Reference
ABP-GB123	B, S, L	NA	NA	NA	[55,58]
ABP-BMV101	B, S, L	Y	Y	Y	[57]
qABP-BMV109	B, S, L, X	Y	Y	NA	[56,59]
qABP-GB137	B, L	NA	NA	Y	[52]
qABP-YBN1-8	B, S, L	Y	Y	Y	[51]
qABP-BMV083	B, S, L	Y	NA	NA	[56]
qABP-BMV117	S	Y	NA	NA	[56]
qABP-BMV157	S	Y	Y	Y	[56]

## Abbreviations

aa	Amino Acids
BC	Breast Cancer
CC	Colon Carcinoma
CRC	Colorectal Cancer
ECM	Extracellular Matrix
KDa	Kilodaltons
LL	Lysosomal Leakage
LMP	Lysosomal Membrane Permeabilization
PCD	Programmed Cell Death
PDAC	Pancreatic Ductal Adenocarcinoma
ROC	Receiver Operating Curve Analysis
